# The Type VI secretion system in enteric pathogen colonization: molecular mechanisms, ecological dynamics, and therapeutic potential

**DOI:** 10.3389/fmicb.2026.1809019

**Published:** 2026-03-10

**Authors:** Chang Sui, Huihuang Qiao

**Affiliations:** 1 Department of Oral Imaging, Stomatological Hospital, Jilin University, Changchun, China; 2 Department of Orthopedics, China-Japan Union Hospital, Jilin University, Changchun, China

**Keywords:** colonization resistance, gut microbiota, host-microbe interaction, interbacterial competition, microbiome engineering

## Abstract

The Type VI Secretion System (T6SS) is a sophisticated, phage-tail-like contractile nanomachine that mediates contact-dependent protein translocation in a wide range of Gram-negative enteric pathogens. As a primary weapon for interference competition, T6SS enables pathogens like *Salmonella* and *Vibrio cholerae* to directly eliminate commensal rivals. This targeted elimination allows pathogens to dismantle microbiota-mediated colonization resistance and seize essential nutritional niches. Beyond interbacterial warfare, the system facilitates “exploitative competition” by secreting effectors for the acquisition of limited micronutrients such as iron and zinc. Furthermore, T6SS acts as a crucial virulence determinant by manipulating host cell signaling, disrupting cytoskeletal integrity, and even enhancing intestinal contractions to physically expel competitors. The expression and activity of T6SS are dynamically regulated by gastrointestinal cues, including bile salts, pH fluctuations, and quorum sensing signals, ensuring its activation is precisely timed during infection. Elucidating these multifaceted roles not only deepens our understanding of microbial ecology in the gut but also highlights T6SS as a promising target for microbiome engineering and the development of customizable, precision antimicrobial therapies.

## Introduction

The human gastrointestinal tract is a densely populated ecosystem that, particularly during pathogen invasion, transforms into an inflamed and hyper-competitive battlefield. In this dynamic environment—where pathogens like Salmonella often exploit host immune responses to gain a fitness advantage—trillions of microorganisms engage in a continuous struggle for space and nutritional resources (Athanasopoulou et al., 2023; Ma et al., 2026). For enteric pathogens, the primary barrier to successful establishment is “colonization resistance”—a multifaceted defense mechanism provided by the indigenous microbiota through nutrient sequestration, production of inhibitory metabolites, and the priming of host innate immunity (Li and Guo, 2026). To overcome this colonization resistance, pathogens such as *Salmonella enterica*, *Vibrio cholerae*, and *Citrobacter rodentium* have evolved a sophisticated arsenal of virulence factors. Among these, the Type VI Secretion System (T6SS) stands out as a prevalent and highly efficient contact-dependent nanomachine, identified in approximately 25% of all sequenced Gram-negative bacteria (predominantly within the Proteobacteria phylum) (Unni et al., 2022; Wohlfarth et al., 2025). Structurally homologous to an inverted bacteriophage tail, the T6SS functions as a nanometer-scale “injection system,” utilizing a contractile sheath to propel a toxin-loaded needle directly into the cytoplasm or periplasm of adjacent target cells (Wohlfarth et al., 2025). This mechanism allows pathogens to engage in “interference competition,” delivering a lethal array of effector proteins—including peptidoglycan hydrolases, phospholipases, and nucleases—to eliminate commensal rivals that occupy their metabolic niches (Dong et al., 2025; Jiang et al., 2025; Zhu et al., 2025).

The functional versatility of the T6SS extends far beyond simple interbacterial warfare, encompassing what microbiologists categorize as *multidimensional colonization strategies*. Beyond direct killing, the T6SS facilitates “exploitative competition” by secreting proteinaceous metallophores that scavenge essential micronutrients, such as iron, zinc, and manganese, from the gut environment where they are often limited by host-mediated “nutritional immunity” (Deshazer, 2019). Perhaps more remarkably, the T6SS serves as a potent “trans-kingdom” weapon capable of subverting host eukaryotic processes to favor pathogen persistence. For instance, certain T6SS effectors in *Vibrio cholerae* contain actin cross-linking domains that disrupt the host cytoskeleton, not only disabling phagocytic defense but also inducing intestinal contractions to physically expel resident microbiota, thereby “remodeling” the community to the pathogen’s advantage (Dutta et al., 2019). This ability to simultaneously suppress microbial competitors and manipulate host biomechanics represents a critical evolutionary adaptation for establishing dominance within the highly regulated intestinal landscape.

The clinical significance of the T6SS is underscored by its remarkable genomic plasticity and its integration into the global virulence networks of enteric pathogens. In *Salmonella*, for example, T6SS clusters are differentially distributed across various pathogenicity islands (SPI-6, SPI-19, etc.), allowing different serotypes to adapt to specific hosts and ecological pressures through horizontal gene transfer (Blondel et al., 2025). To prevent self-intoxication, these systems are organized into functional “effector-immunity” (E/I) modules, where each toxic effector is paired with a cognate immunity protein (Blondel et al., 2025). This modular architecture dictates the competitive fitness of a strain in polymicrobial environments. As traditional antibiotics continue to face challenges from rising resistance, the T6SS offers a promising frontier for the development of “precision antimicrobials” and “microbiome engineering.” By deciphering the molecular cues—such as bile salts, pH, and quorum sensing—that trigger T6SS activation, and by harnessing its specificity for targeted bacterial killing, researchers are now exploring ways to arm beneficial commensals or design T6SS-based nanoparticles to selectively eradicate pathogens while preserving gut homeostasis. This review provides a comprehensive synthesis of the latest research on the T6SS, examining its structural assembly, its diverse effector repertoire, and its transformative role in the ongoing contact-dependent antagonism of the human gut.

### The molecular architecture and assembly of the T6SS

The T6SS is an expansive protein complex that spans the bacterial envelope, characterized by a structural logic that bridges the gap between static transport machinery and dynamic contractile weapons (He et al., 2023). Its assembly follows a strictly hierarchical path, beginning with the formation of the membrane complex (MC), which acts as a stable anchor and an iris-like gate (Zoued et al., 2014). In Proteobacteria, this 1.7 MDa complex is composed of ten units of a heterotrimer containing TssJ, TssM, and TssL (Zoued et al., 2016). TssM is a core scaffold protein that spans the inner membrane and connects to the outer membrane lipoprotein TssJ, while TssL provides further stability by interacting with the baseplate (Zoued et al., 2014). Unlike the tail and baseplate components, which share extensive structural homology with contractile bacteriophages, the specific proteins comprising the membrane complex (TssJ, TssM, and TssL) are unique to the T6SS machinery. They serve as the specialized anchor that integrates the bacteriophage-like apparatus into the bacterial envelope. The baseplate (BP), comprising proteins such as TssE, TssF, TssG, and TssK, then assembles onto the MC and serves as the foundation for the polymerization of the tail (Zoued et al., 2014). The baseplate functions as a molecular checkpoint for cargo occupancy. Recent structural studies suggest that specific interactions between effectors (often aided by cognate chaperones) and the VgrG/PAAR spike complex are required to stabilize the baseplate hub. This stabilization serves as a structural prerequisite for the polymerization of the tail sheath, thereby preventing the energy-intensive assembly and firing of “empty” T6SS particles and preserving metabolic fitness.

The most visually striking component of the T6SS is the tail tube/sheath complex (TTC), a dynamic tubular structure that can reach lengths of up to 600 nm, effectively spanning the bacterial cytoplasm (Basler et al., 2012). The inner tube is built from stacked hexameric rings of the hemolysin co-regulated protein (Hcp), which also serves as a chaperone for various cargo effectors (Cherrak et al., 2019). This tube is topped by a trimeric spike of the Valine-Glycine Repeat protein G (VgrG), which is further sharpened by a pointed Proline-Alanine–Alanine-Arginine (PAAR) domain protein (Renault et al., 2018; Cherrak et al., 2019). Surrounding this inner core is the contractile sheath, formed by the polymerization of TssB and TssC (VipA/VipB) subunits (Cherrak et al., 2019). The assembly of the inner tube and the sheath is interdependent and coordinated by the dodecameric TssA complex, which sits at the growing tip of the tail to guide ring addition (Cherrak et al., 2019). Once the tail reaches the opposite cell membrane or a predetermined length, the assembly is stopped—potentially by “stopper” proteins like TagA—and maintained in a metastable, high-energy state (Stietz et al., 2020). Upon triggering by environmental cues or physical contact, the sheath undergoes a rapid, conformational change, contracting and driving the inner tube and spike into the target cell with enough force to penetrate both prokaryotic and eukaryotic membranes. Following firing, the ATP-dependent AAA + ATPase ClpV disassembles the contracted sheath, recycling the subunits for subsequent rounds of attack (Cherrak et al., 2019).

### Diversity and function of the T6SS effector arsenal

The competitive success of T6SS-carrying pathogens is ultimately dictated by the biochemical diversity of the effectors they deploy. These effectors are categorized into “specialized” (or evolved) effectors, which are covalently fused to the structural components Hcp, VgrG, or PAAR, and “cargo” effectors, which associate non-covalently with the apparatus (Blondel et al., 2025), sometimes requiring specific chaperones or adaptors for loading (Li W. et al., 2025). The diversity of these toxins is immense, targeting nearly every essential feature of a rival bacterial cell. Periplasmic-acting effectors typically target the cell wall or membrane; for instance, the Type VI Amidase Effector (Tae) family and Type VI Glycoside Hydrolase (Tge) family degrade peptidoglycan, leading to rapid cell lysis (Le et al., 2021; Bezkorovayna et al., 2025). Membrane-targeting effectors include phospholipases (Tle superfamily) and pore-forming toxins that disrupt electrochemical gradients, such as VasX in *V. cholerae* (Miyata et al., 2011; Ren et al., 2023). Cytoplasmic effectors are even more varied, attacking core macromolecules such as DNA (DNases), RNA (RNases), and essential metabolic cofactors like NAD + and NADP+ (Tang et al., 2018; Luo et al., 2023; Sun et al., 2024; Wang Y. et al., 2025).

A burgeoning area of T6SS research involves “trans-kingdom” and “exploitative” effectors, which expand the pathogen’s reach beyond simple bacterial killing. Trans-kingdom effectors can intoxicate both prokaryotic and eukaryotic cells by targeting conserved features like the cytoskeleton or genetic material. For example, the dual-targeting DNase TkeA in *Yersinia pseudotuberculosis* promotes bacterial antagonism while simultaneously inducing host cell apoptosis via the cGAS-STING-TNF axis (Wang Y. et al., 2025). Exploitative effectors are secreted to capture scarce environmental resources, particularly metal ions like zinc and manganese, which are sequestered by host “nutritional immunity.” Effectors such as YezP in *Y. pseudotuberculosis* and TseM in *Burkholderia thailandensis* act as proteinaceous metallophores, binding extracellular ions and returning them to the cell via specific outer membrane receptors (Si et al., 2017; Liu et al., 2023). This mechanism not only sustains the pathogen’s growth but also starves competitors of essential nutrients. To survive this internal toxicity, T6SS-producing bacteria express cognate immunity proteins that bind to and neutralize their own effectors, ensuring that only “non-kin” cells are eliminated (Blondel et al., 2025).

### Ecological impact and microbiota remodeling during colonization

In the gut, the T6SS is a central modulator of community assembly, allowing pathogens to establish ecological niches by forcibly displacing established commensals. The “interference competition” mediated by the T6SS is particularly critical during the early stages of infection when the pathogen is most vulnerable to colonization resistance (Serapio-Palacios et al., 2022; Jiang et al., 2024; Bray et al., 2025). *Salmonella Typhimurium*, for instance, uses its SPI-6 encoded T6SS to eliminate commensals like *Klebsiella oxytoca*, with whom it shares a high degree of metabolic overlap for simple sugars (Sana et al., 2016). By neutralizing these competitors, *Salmonella* can occupy the specific nutritional niches necessary for its expansion in the inflamed gut. Constitutive T6SS expression in *Vibrio cholerae* has similarly been shown to provide a significant advantage in both inter-specific and intra-specific competition, facilitating its survival and dominance during host colonization (Logan et al., 2018; Zhao et al., 2018; Breen et al., 2021b). Beyond antibacterial warfare, the T6SS facilitates interkingdom surveillance; *Yersinia pseudotuberculosis* senses the fungal quorum-sensing molecule tyrosol via the EnvZ-OmpR system, triggering the secretion of the antifungal chitinase TfeC to eliminate *Candida albicans* and reduce fungal prevalence (Zhu et al., 2026). Interestingly, in some models, the T6SS does not just kill rivals but reshapes the community through host biomechanical manipulation; *V. cholerae* utilizes its VgrG-1 actin cross-linking domain (ACD) to increase the strength of intestinal contractions in zebrafish, physically expelling resident species and clearing the way for its own population (Ma and Mekalanos, 2010; Breen et al., 2021a).

Furthermore, T6SS-mediated killing has profound evolutionary consequences by driving horizontal gene transfer (HGT). When a T6SS-positive bacterium lyses a rival, the released DNA becomes available for uptake by naturally competent cells in the vicinity. This bacteriolysis-coupled transformation strategy has been observed in *Vibrio cholerae* and *Acinetobacter baylyi*, where the T6SS is co-regulated with natural competence machinery, allowing for the rapid acquisition of antibiotic resistance or virulence genes from the surrounding community (Ringel et al., 2017; Thomas et al., 2017). This highlights the T6SS not just as a weapon of destruction, but as a catalyst for genetic diversification and adaptation within the gut microbiome. Conversely, the T6SS can also play a stabilizing role in beneficial commensals; *Bacteroides fragilis* utilizes three distinct T6SS architectures (GA1-GA3) to maintain its ecological stability and dominance in the human gut, even providing protection against gastrointestinal acute graft-versus-host disease (GI-aGVHD) by modulating bile acid metabolism and preventing the accumulation of pro-inflammatory primary bile acids (Li P. et al., 2025).

### Regulatory control: sensing the gut microenvironment

The assembly and firing of the T6SS are under stringent regulatory control, ensuring that this energetically expensive system is only activated when it provides a competitive advantage. This regulation is integrated with the sensing of both abiotic and biotic cues characteristic of the gastrointestinal tract. Abiotic factors such as pH and osmolarity are primary signals; for example, the T6SS in *V. cholerae* O1 is repressed at low osmolarity but upregulated under high osmolarity and warm temperatures that mimic its natural estuarine and host habitats (Shao and Bassler, 2014). Recent research has revealed that this environmental sensing extends to specific host-derived and pharmacological cues. In *Klebsiella pneumoniae*, the PhoPQ two-component system, typically associated with Mg^2+^ sensing, has been shown to activate T6SS expression in response to host antimicrobial peptides (such as human defensin 3) and antibiotics like polymyxin B (Storey et al., 2020). Conversely, exposure to sub-inhibitory concentrations of aminoglycosides can trigger the regulator AmgR in *Pseudomonas aeruginosa* to repress the H1-T6SS, orchestrating a switch from chronic-associated T6SS activity to acute virulence phenotypes (Wang X. et al., 2025). Diet also imposes selective pressures; high-protein diets rich in casein can downregulate *V. cholerae* T6SS expression via the central flagellar regulator FlrA, reducing competitive fitness (Liu et al., 2025). Beyond specific nutrients, host feeding patterns have been found to reprogram the gut microbial virulence-iron-quorum sensing axis, thereby linking bacterial offensive behaviors to host systemic health risks such as atherosclerosis (Zhang et al., 2025). Furthermore, while mucins generally activate the *V. cholerae* T6SS, specific secondary bile acids like deoxycholic acid can counteract this activation, effectively dampening killing efficiency (Bachmann et al., 2015).

**Figure 1:**
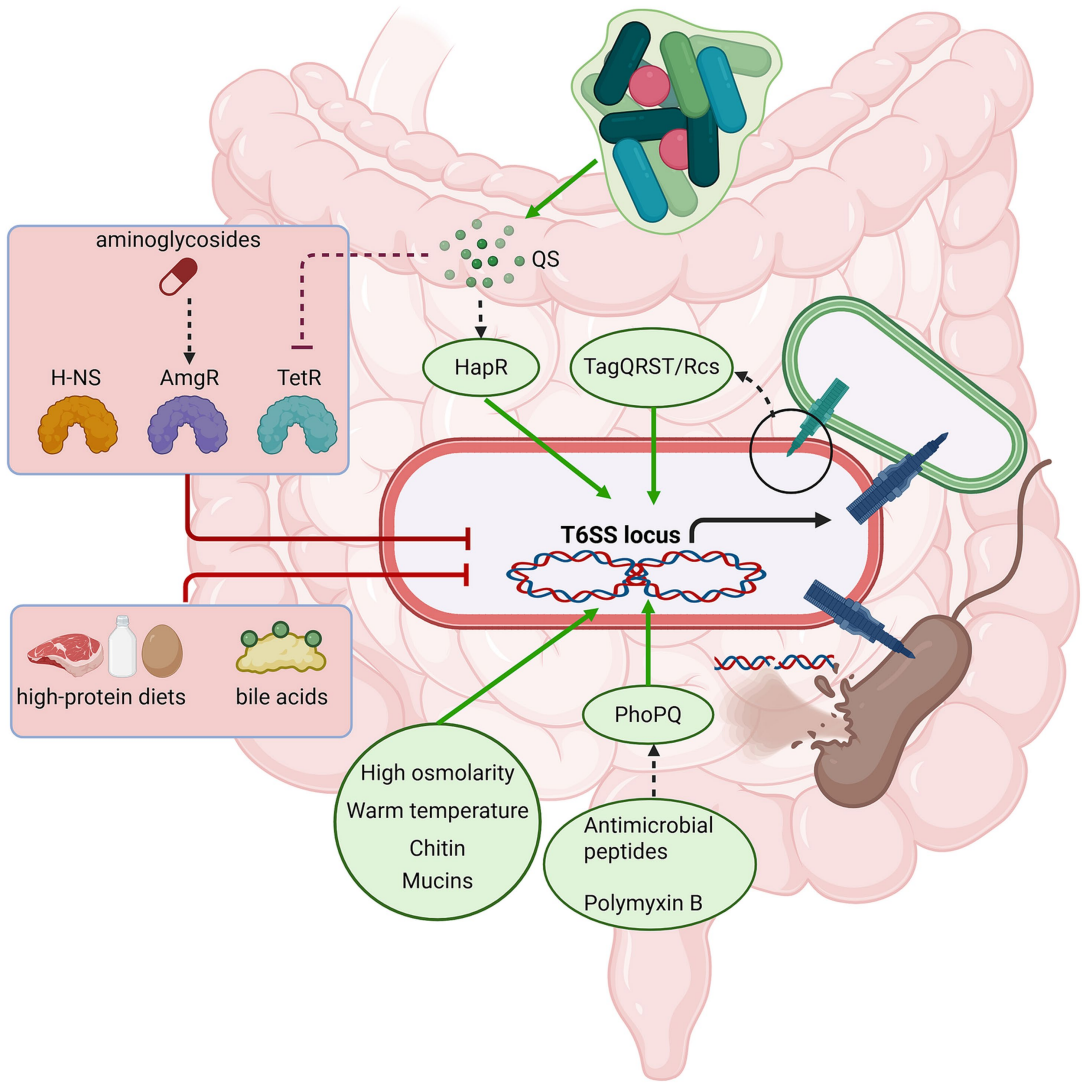
Integrated regulatory network governing T6SS activation and repression. The diagram illustrates a T6SS-armed pathogen integrating multimodal signals within the gut microenvironment. Green arrows indicate activation pathways, while red T-bars indicate repression. Environmental factors (high osmolarity and temperature) and host-derived mucins act as activating signals. In contrast, specific secondary bile acids and sub-inhibitory antibiotics serve as repressing signals, with bile acids specifically counteracting mucin-dependent activation. High cell density within the bacterial population activates the T6SS via quorum sensing (QS) mechanisms. Physical attacks from rival bacteria cause cellular damage, which is rapidly detected by the pathogen to trigger an immediate retaliatory T6SS firing response.

Biotic signals, including quorum sensing (QS) and the detection of “hostile” microbial activity, also play a vital role. In *V. cholerae*, T6SS expression is dependent on the QS regulator HapR, which synchronizes the population’s offensive behavior at high cell densities. In the commensal *Bacteroides fragilis*, the pathogenicity island (BFPAI) serves as a critical nexus physically and functionally linking virulence determinants with strain competition machinery (Casterline et al., 2017); this system is strictly controlled by a TetR-family repressor that responds to a self-produced small molecule ligand, ensuring activation only under specific population contexts (Tuzlak et al., 2025). Some bacteria, like *P. aeruginosa*, have evolved a sophisticated “defensive” or “retaliatory” firing mechanism (Basler et al., 2013). Through the TagQRST sensor module, *P. aeruginosa* can sense membrane damage or mechanical stimulus from a rival’s T6SS attack and promptly trigger its own H1-T6SS to strike back. Similarly, *Serratia marcescens* employs the Rcs phosphorelay system as a “transcriptional rheostat” that finely tunes T6SS expression specifically proportional to the cellular damage caused by incoming effectors from aggressive competitors (Lazzaro et al., 2017). This “dueling” behavior allows the bacterium to resist incoming assaults and maintain its position in polymicrobial biofilms. Additionally, the presence of host-derived mucins and chitin (relevant for marine-associated pathogens) has been shown to positively regulate T6SS activity and induce natural competence, effectively coupling bacterial killing with DNA uptake for horizontal gene transfer (Blokesch, 2015), further illustrating how pathogens tune their secretion machinery to the specific biological landscape they inhabit (Figure 1).

### Host-pathogen interaction and immune subversion

Beyond microbial warfare, the T6SS serves as a classical virulence factor by delivering toxins that directly subvert host eukaryotic cells. This aspect of the T6SS allows pathogens to manipulate cellular processes such as adhesion, internalization, and the evasion of innate immune responses. As previously mentioned, the *Vibrio cholerae* VgrG-1 ACD induces the covalent cross-linking of actin monomers, leading to cytoskeletal collapse, cell rounding, and the inhibition of phagocytosis (Ma and Mekalanos, 2010; Breen et al., 2021a). Recent findings emphasize the T6SS’s versatility in manipulating cytosolic surveillance pathways. For instance, pathogens can subvert host defenses by sequestering manganese—a critical cofactor for the DNA sensor cGAS—thereby suppressing cGAS-STING signaling (Zhu et al., 2021). Conversely, the T6SS can actively drive programmed cell death; a dual-targeting T6SS DNase has been shown to induce eukaryotic apoptosis via the cGAS-STING-TNF axis, highlighting a mechanism where bacterial antagonism and host toxicity are coupled (Song et al., 2025). While some pathogens suppress inflammation to evade detection, others exploit it. By selectively modulating these pathways, the pathogen can attenuate the host’s early immune response or induce specific cell death modes to promote dissemination.

The interaction between the T6SS and host immunity is complex and can sometimes involve “accidental” activation of host defenses. The *E. piscicida* effector Trxlp promotes NLRC4 inflammasome activation (Xu et al., 2018), and *V. proteolyticus* effectors Tie1 and Tie2 trigger post-phagocytosis NLRP3 assembly (Cohen et al., 2022). However, pathogens often evolve compensatory mechanisms to manage this risk. *Edwardsiella tarda* secretes the effector EvpP, which inhibits the NLRP3 inflammasome by suppressing the Ca^2+^-dependent MAPK-Jnk pathway (Chen et al., 2017). In the context of beneficial symbionts like *Snodgrassella alvi* in the honey bee gut, the T6SS has been shown to modulate the expression of host antimicrobial peptides (e.g., apidaecin), suggesting that the system may play a broader role in host-microbe “immunological conditioning” (Motta et al., 2024). These findings indicate that the T6SS is not just a weapon but a sophisticated interface for trans-kingdom communication, capable of modulating the dynamics of host-microbe homeostasis to suit the pathogen’s or symbiont’s survival strategies.

### Clinical implications and synthetic biology applications

The profound role of the T6SS in determining microbial fitness makes it an attractive target for novel antimicrobial strategies and microbiome engineering. One promising approach is the development of “precision antimicrobials” that target the T6SS machinery itself. By identifying small molecules that inhibit core assembly components like the ClpV ATPase or the TssM membrane complex, researchers could effectively “disarm” pathogens without killing them, thereby reducing the selective pressure for antibiotic resistance. However, the discovery of such inhibitors requires robust screening platforms beyond traditional phenotypic assays. To this end, recent methodological advances, such as the development of a quantitative luciferase-based reporter system, have overcome the low throughput of bacterial killing assays (Yang et al., 2025). Adopting similar high-sensitivity, fluorescence-based strategies for enteric pathogens would significantly accelerate the screening of clinical isolates and potential anti-virulence drugs. Furthermore, the diverse and potent array of T6SS toxins represents a rich reservoir of naturally validated antibacterial activities that could be adapted into new classes of antibiotics (Le et al., 2021; Luo et al., 2023).

Synthetic biology offers an even more radical possibility: the engineering of smart, T6SS-based “living therapeutics.” Researchers have already demonstrated a proof-of-concept by introducing an inducible and customizable T6SS platform into the safe, non-pathogenic marine bacterium *V. natriegens* (Jana et al., 2021). By swapping the delivered effector-immunity modules, this platform can be tailored to selectively kill specific multi-drug resistant pathogens, such as *E. coli* or *C. difficile*, while leaving the rest of the microbiota intact (Jana et al., 2021). Such “bacteria-on-bacteria” therapy could provide a highly targeted alternative to broad-spectrum antibiotics, which often cause collateral damage to the protective commensal community. Moreover, the engineering of commensals to be immune to pathogen T6SS attacks—by equipping them with the relevant immunity proteins—could reinforce colonization resistance and prevent infection before it starts (Keith and Pamer, 2019). As our understanding of the T6SS’s “molecular rules” deepens, we are moving toward an era of precise microbiome editing, where we can actively manage the interbacterial interactions of the gut to promote human health.

## Conclusion

The Type VI Secretion System is a cornerstone of enteric pathogen survival, representing one of nature’s most sophisticated and versatile molecular machines. From the precise structural logic of its phage-like tail to the immense biochemical diversity of its effector arsenal, the T6SS provides a multidimensional advantage that allows pathogens to breach colonization resistance, capture scarce nutrients, and subvert host defenses. Its role in shaping the gut microbiome through both interference and exploitative competition, as well as its capacity to drive horizontal gene transfer, positions it as a primary architect of microbial ecology. For the microbiologist, the T6SS is no longer just a “toxin delivery system,” but a complex sensing and communication hub that defines the boundaries between health and disease. As we transition from observation to intervention, harnessing the power of the T6SS offers a revolutionary pathway toward precision medicine, microbiome engineering, and the development of intelligent, bio-inspired therapies for the post-antibiotic era. The ongoing study of this “molecular crossbow” continues to unveil the extraordinary depths of bacterial evolution and its profound impact on human host-pathogen dynamics.
